# High Performance Field Emitters

**DOI:** 10.1002/advs.201500318

**Published:** 2016-02-18

**Authors:** Clare M. Collins, Richard J. Parmee, William I. Milne, Matthew T. Cole

**Affiliations:** ^1^ Department of EngineeringElectrical Engineering DivisionUniversity of Cambridge9 JJ Thomson AvenueCB3 0FACambridgeUK; ^2^ Quantum Nanoelectronics Research CentreTokyo Institute of Technology152‐8550TokyoJapan

**Keywords:** 2D materials, aspect ratio, field electron emission, field enhancement, nanomaterials, work function

## Abstract

**The field electron emission performance of bulk, 1D, and 2D nanomaterials** is here empirically compared in the largest metal‐analysis of its type. No clear trends are noted between the turn‐on electric field and maximum current density as a function of emitter work function, while a more pronounced correlation with the emitters dimensionality is noted. The turn‐on field is found to be twice as large for bulk materials compared to 1D and 2D materials, empirically confirming the wider communities view that high aspect ratios, and highly perturbed surface morphologies allow for enhanced field electron emitters.

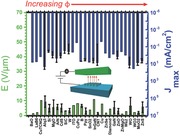

Cold cathode field emission from nanomaterials is an on‐going area of great academic and technological interest. There have been many suggested applications of field electron emission, including displays,^1^, ^2^, ^3^ traveling wave tubes,^4^, ^5^ microwave amplifiers,^6^, ^7^ electron microscopy,^8^, ^9^ parallel electron beam lithography,^2^, ^10^, ^11^ and X‐ray sources.^2^, ^12^, ^13^ Low work functions have been repeatedly touted as one of the primary drivers towards achieving high performance field electron emission.^14^, ^15^ However, detailed analysis of the way in which the work function affects the field emission has not yet been fully understood or comprehensively studied across a diverse range of materials. In the case of conventional bulk metallic systems, low work functions result in higher current densities relative to those materials with high work functions, as suggested by the established Fowler–Nordheim theory. As a result of this widely, and perhaps often incorrectly adopted theory, many have strived to develop low work function materials, composites, or coatings for enhanced field emitters.^16^, ^17^, ^18^ Empirical confirmation of the relative merits of low work function is, however, lacking. In this work, the effect of the work function and emitter dimensionality are studied in the largest meta‐analysis of its type.

A considerable amount of data is available from a broad range of materials, which has been considered as viable candidates for field emission. No one, to date, however, has attempted to draw direct comparisons between said materials. Only pure materials are considered herein; all adlayers and materials with surface coatings have been intentionally excluded from the present study for simplicity. Ease of comparison between a range of materials allows for a comprehensive understanding of which materials are most suited for the use in various field emission applications. Differing field emission applications call for widely disparate electron emission performance, and a comparative knowledge of the available materials suited to said applications is technologically critical. In this paper, we show by considering the breadth of materials from published literature that an electron emitter's work function does not significantly influence the field emitting capabilities of a material, when the work function is the only comparative characteristic.

Here the studied materials are classified into categories according to dimensionality; namely, 1D, 2D, and 3D/bulk materials. The materials deposited or grown on the substrate function as a field electron emitting cathode (**Figure** **1**a). The liberated electrons tunnel through the restraining surface potential, whatever profile this may adopt, into ultrahigh‐vacuum conditions and are subsequently accelerated toward the anode. 1D materials are characterized by very high aspect ratios with nominal widths at the nanometer scale and typical lengths of at least one order of magnitude longer than their width. 1D emitters are diverse in structure, though often consist of aligned or disordered forests of 1D nanowires (NWs), which may be patterned, using conventional lithographic techniques, where the density of the 1D materials can be controlled either by the detailed growth conditions or the number of deposition cycles. 2D materials include the graphenes: a single sheet of hexagonally latticed carbon atoms, as well as the broader family of transition metal dichalcogenides. All are atomically thin, with typical single grains ranging in diameter from a few tens of nanometers, to many hundreds of micrometers. These layers may be regularly stacked, though more often adopt a more disordered morphology. All 2D materials studied here were polycrystalline, and were either grown directly, or deposited additively on various substrates, via processes much like those employed for the 1D nanomaterials. Bulk emitters often have microcone geometry. They possess structures that consist of complex atomic and macroscopic arrangements; they can be crystalline, amorphous, disordered, or structured. Nevertheless, their primary defining trait is their characteristically low aspect ratio, which is typically <10. A number of materials in each of these categories have been used in field emission studies, with a particularly large number within the 1D set, attributed to an increase in interest in nanowires and nanotubes in recent history, and the sharp tips that they offer.

**Figure 1:**
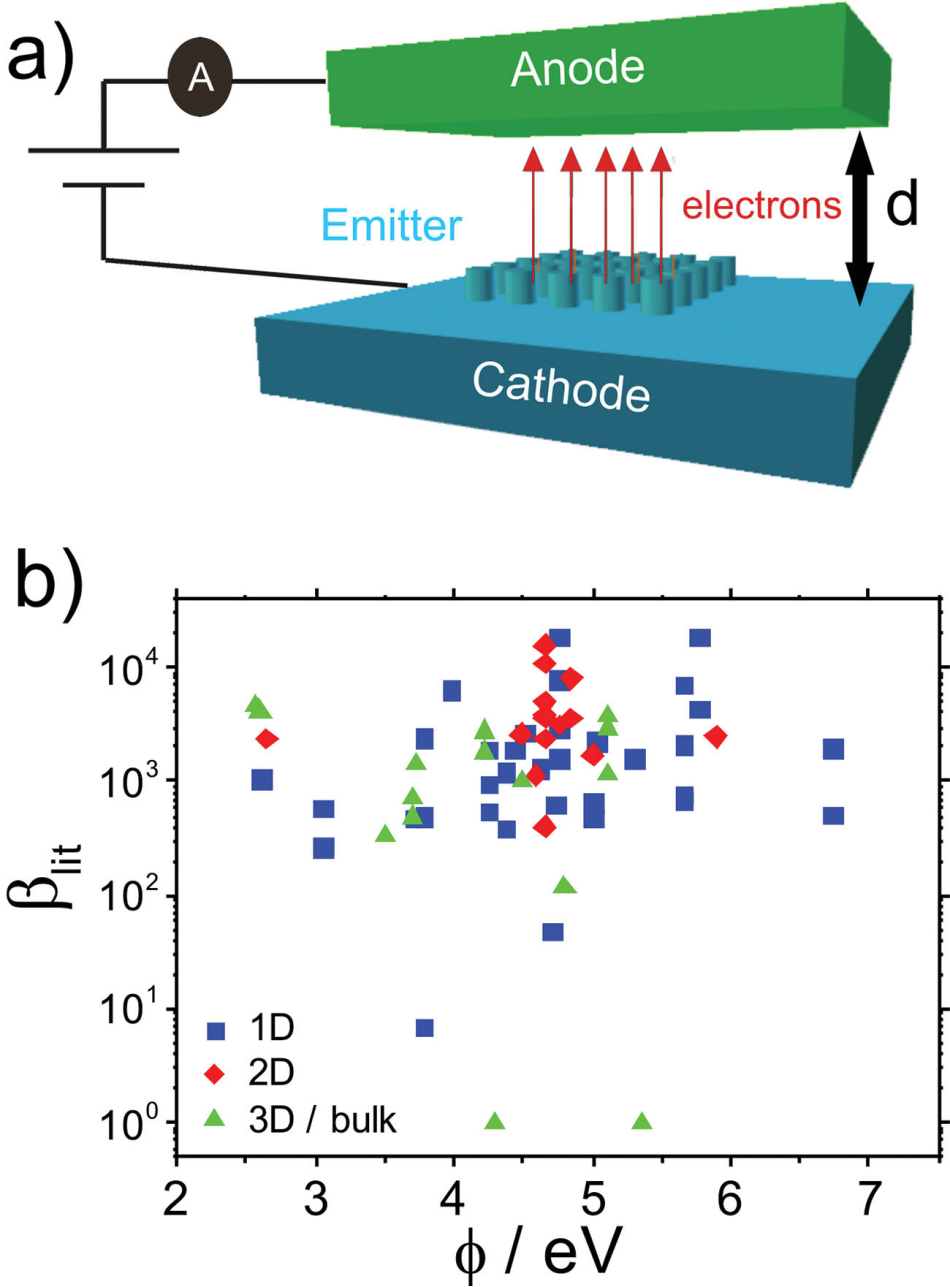
a) Generalized field emitting device. All studies considered herein use exclusively diode mode operation. b) Emitter field enhancement factor (*β*) against work function (*φ*) for 1D, 2D, and 3D/bulk materials, with little dependency seen.

The key parameters for the success of a material as a field emitter are a low turn on electric field, *E*_on_, a low threshold electric field, *E*_thr_, and a high maximum current density, *J*_max_. Though key in assessing the emission performance, these metrics have been, to date, poorly defined. They vary dramatically between publications,^19^, ^20^, ^21^, ^22^ with many seemingly almost arbitrary definitions. Just under half of the papers studied herein reported values for *E*_on,_ usually defined at an emission current density of 0.01 mA cm^−2^. *E*_thr_ is stated less frequently (20% of papers studied), at common current densities of 0.1, 10, and 1 mA cm^−2^. Historically, the emission current density required to visualize electron emission patterns on phosphorescent screens was given as 10 nA cm^−2^.^23^ 10 mA cm^−2^ is widely quoted as a “figure of merit,” particularly with regards to flat panel displays, though with no clear reasoning is given as to why.^24^, ^25^ The use of the commonly reported values in other field emission applications appears undefined, however, and they are not exclusively quoted. Indeed, some acknowledge that there are no strict rules, with some groups opting to define their own metrics.^26^, ^27^, ^28^, ^29^ Due to such arbitrary definitions and the apparent lack of consistency, it has proven, to date, prohibitively challenging to draw direct valid comparisons between differing materials and morphologies.

In order to compare materials, a new definition was tested. Figure 1a shows a generalized field emitting device operated in diode mode. The emitting material, located in high vacuum conditions, is negatively biased and exposed to a high voltage, typically of the order of a few thousand volts on the anode. The interelectrode vacuum gap (d) defines the apparent global electric field. Here we adopt more generalized definitions for *E*_on_ and *E*_thr_, defining them as 10% and 30%, respectively, of a normalized total measured current density, *J*′ = /*J*_max_, when subjected to an applied electrostatic field (*E*). Data extracted in this way, across a breadth of studies, assuming consistency in *J*_max_, can then be directly compared, allowing for the largest study to date across a near exhaustive range of materials. Data were extracted from 112 published papers dating from 1984 to the present day. Where more than one paper per material was found the extracted *E*_on_ and *E*_thr_ were averaged. Some materials, such as carbon nanotubes (CNTs) and graphene, proved common place, whereas with other, less popular materials, such as FePc 30 and cBN,^31^ only a single paper was available. Work functions are averaged throuout (<*n*> ≥ 3). Having redefined the parameter *E*_on_, we noted that extrate E_on_ depends critically on *J*_max_ , highlighting a clear deficiency with this appraoch. *E*_on_ directly relates to *J*_max_, therefore altering with overall performance figure of merit. Whilst this generalised approach allowed for direct comparisons to be made between materials, a similar comparison can be made by simply defining *E*_on_ as a single value of current density. We stress, however; that the two methodologies resulted in similar data sets; though we have nonetheless opted for the latter approach given its simplicity and consistency with existing literature. The most commonly used current density used in the literature to define E_on_ was 0.01 mA cm^−2^, which largely justifyies this otherwise arbitrary figure. Not all materials could be measured, this was due to the range of measurements made individually; however, this was only around 2% of the studies considered.

In almost all studies to date, the measured FE properties have been mostly well‐fitted with conventional Fowler–Nordheim tunneling, as given by

(1) $$J\;=\;\left(\frac{A\beta^{2}E^{2}}{\varphi }\right)\exp\left[\frac{-B\varphi^{\frac{3}{2}}}{\beta E}\right]$$

where *J* is the current density, *A* = 1.54 × 10^−6^ A eV V^−2^ is a constant, *φ* is the emitter work function, *B* = 6.83 V eV^3/2^ cm^−1^ is a constant, and *E* is the applied electric field. The electric field, *E*, can be approximated using the anode‐cathode voltage (V) and inter‐electrode separation (*d*) by E = β(V/d), where *β* is the local field enhancement factor. The validity of the Fowler–Nordheim theory across most material platforms is certainly questionable, especially for materials that are not classical bulk metals.^32^ Nevertheless, the emission current dependence on the materials work function has been widely implicated in various tunneling models, as has the aspect ratio, or degree of perturbation in the emitting material. Nonetheless, the degree of suitability of models, such as Fowler–Nordheim, for materials where the tip radius of curvature is less than a few tens of nanometers^33^ is still yet to be determined with any great accuracy. It can be seen that *J*, and hence *J*_max_, can be tuned by augmenting *φ* and *β*, both of which can be altered by the surface geometry and chemistry. According to the general WKB approximation and subsequent transmission models based thereon, low *φ* and high *β* typically manifest as high *J*_max_. In practice, however, for non‐classical materials, such as nanowires and nanotubes, the intimate mechanisms which augment the emission are not yet fully understood.^34^ This study focuses on the effect of changing φ across a diverse material range in an attempt to rationalize the importance of emitter work function in comparison to the degree of perturbation in emitter geometries.

The extracted performance metrics (*E*_on_, *J*_max_) are organized according to the work function (*φ*), from lowest to highest. Another factor that is commonly implicated in affecting the field emitting performance of a material is the field enhancement factor (*β*). *β* relates the local electric field surrounding the emitter apex (*E*_0_) to the linearly approximated macroscopic electric field (*E*), where $\beta \;=\;\frac{E_{0}}{E}$. Around 70% of the papers studied reported *β* , highlighting another inconsistency in the field. *β* is poorly defined, with some quoting it as the value of height (*h*) of the emitter over the radius of curvature (*r*) of the tip: (h/r),^35^, ^36^ or some linearly scaled variation of this, with this scalar varying between 1^27^, ^37^, ^38^, ^39^ and 25.^28^ Others, more commonly (as is the case for all the 1D materials studied herein, and over 50% for 2D and 3D) state a value of *β* calculated by extracting it from a selected gradient on their coarsely fitted Fowler–Nordheim data. Some (7.5% of all papers studied) provide an empirical validation of such values by comparing them with *β* estimates using other methods, such as morphology estimates from electron microscopy imagery.^28^, ^38^, ^40^ Others (2.5% of all papers studied) simply quote a value and suggest that *β* is a result of a combination of the emitter geometry; such as aspect ratio, surface roughness, the size of the vacuum gap, crystal structure, and spatial distribution of the emitters.^41^, ^42^, ^43^ It is not known, nor is any attempt made herein, to understand in these cases, how each of these contributions affects *β* or indeed the emission properties. For clarity, **Table 1** (Supporting Information) shows an exhaustive list of definitions of *β* reported throughout the literature.

Whether there exists a relationship between *φ* and *β* requires further study. Figure 1b suggests that *β* from the literature, herein termed *β*_lit_, does not appear to be a function of *φ* across the range of materials studied. Figure 1b highlights that the qualities most desired and strived for, and hence most commonly reported are low *φ* and high *β*, where a significant proportion of the data points lie at the top, with high *β*, and to the left of the figure, with *φ* < 5.0 eV. 1D materials show the largest spread in *φ*, whereas 2D materials are mostly confined to 4.0–5.0 eV, as they are at present predominately limited to the graphenes or other carbon based materials. 3D materials, on average, show a lower *φ*, reflecting the maturity of these materials, but also a lower *β* than both 1D and 2D. A clear relationship cannot be seen between *φ* and *β*, although it is possible that *φ* can directly affect *β* (and vice versa), where *φ* (*β*) values are used to define *β* (*φ*) using the Fowler–Nordheim slope method: $\beta \;=\;-\left(\frac{b\phi^{\frac{3}{2}}}{k}\right)$ (**Table 1**, Supporting Information**).**

Ordering the extracted *E*_on_ and *J*_max_ performance metrics as a function of increasing *φ* highlights the dependency of the material properties on the field emission performance. **Figure** **2** compares materials ordered by *φ* only, with no consideration to *β* or the surface morphology of the emitter. For each material the standard errors (<*n*> ≃ 3, extracted from literature) are shown. The materials considered include the 1D nanowires – BaO,^44^ LaB_6,_ 43 Copper tetracyanoquinodimethane (CuTCNQ),^45^ Alq3,^40^, ^46^, ^47^ Si,^37^, ^48^, ^49^, ^50^, ^51^ MgO,^52^, ^53^ AlN,^54^, ^55^ CdS,^20^, ^56^, ^57^, ^58^ SiC,^59^, ^60^ W,^61^, ^62^ ITO,^63^ CuPC,^64^ B,^65^, ^66^ PPy,^67^, ^68^, ^69^ SnO_2_,^70^ InGaN,^28^, ^71^, ^72^ CNTs,^21^, ^73^, ^74^, ^75^, ^76^, ^77^ Cu, 78, 79, 80 ZnSe,^81^ diamond,^82^ GaN,^83^ ZnO,^37^, ^84^, ^85^, ^86^, ^87^, ^88^, ^89^, ^90^ ZnMgO,^91^ WS_2_,^92^ WO,^93^, ^94^ WO_3_,^95^ MoO_2_,^96^, ^97^ and ZnS^37^, ^42^, ^98^, ^99^, ^100^, ^101^, the 2D platelets – CuO,^102^, ^103^ h‐BN,^104^, ^105^, ^106^, ^107^ CbO,^108^ MoS_2_,^109^, ^110^ graphene (monolayer, vertically standing, clustered, and few layer),^22^, ^111^, ^112^, ^113^, ^114^, ^115^, ^116^ RGO,^113^, ^117^ C nanowall,^118^, ^119^ WS_2_‐RGO,^120^ ZnO 121 and SnS_2_ 122, 123, 124, and the 3D/bulk materials – *a*‐diamond,^87^, ^125^ LaB_6_,^36^, ^39^, ^43^, ^126^ nanodiamond,^127^, ^128^ DLC,^38^, ^129^ *a*‐C,^29^, ^130^ AlN,^131^ *ta*‐C,^132^, ^133^, ^134^, ^135^ Si tips,^35^, ^136^ ZnSe,^81^ diamond,^137^, ^138^ Cu tips,^79^, ^80^, ^139^ ZnO,^88^, ^140^ Ni tips,^141^, ^142^, ^143^ chemical vapor deposition (CVD) diamond,^144^, ^145^, ^146^ and cBN.^31^, ^147^

**Figure 2:**
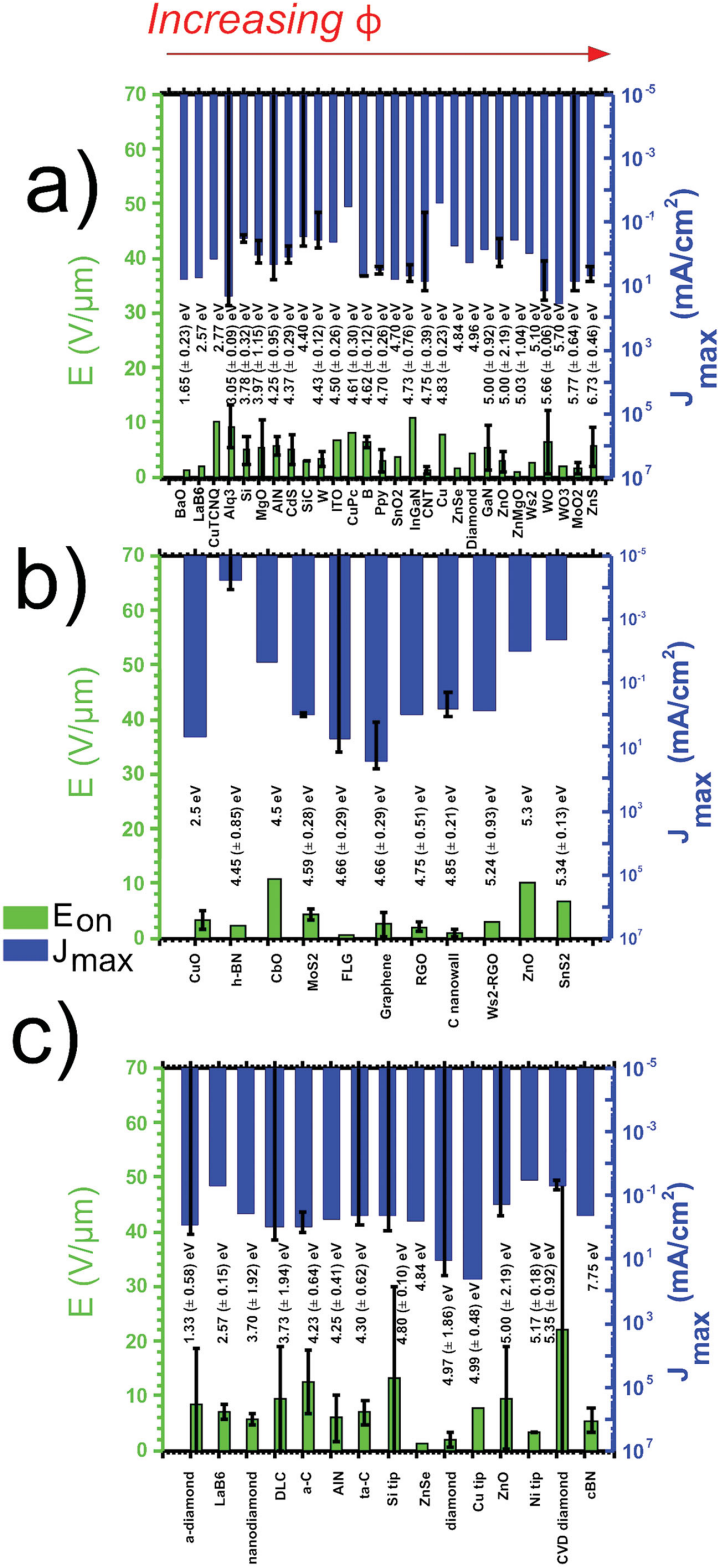
*E*_on_ (green) and *J*_max_ (blue) for a) 1D, b) 2D, and c) 3D/bulk materials ordered by increasing work function (written above material). No distinctive trends were noted, suggesting that work function (*φ*) does not influence, to any great extent, a materials field emitting performance.

Comparisons can easily be drawn between materials in Figure 2 when displayed in this way, both in regards to *φ* and on a material‐to‐material basis. It summarizes a variety of field emission materials, considered across an intentionally diverse range of emitter geometries and morphologies thereby allowing for a valid comparison of intrinsic material properties. When categorized by dimensionality it can be seen that the 2D and 1D materials have very similar average performance (1D: <*E*_on_> | <*J*_max_> = 4.66 V μm^−1^ | 4.85 mA cm^−2^, 2D: 4.21 V μm^−1^| 3.31 mA cm^−2^). 3D/bulk materials, on average, show turn‐on fields approximately twice that of the 1D and 2D sets (3D: <*E*_on_> = 8.09 V μm^−1^). This is likely due to the sharp vertices in 1D and 2D materials, with sizes down to the atomic range in some cases. A very similar average <*J*_max_> (3D: <*J*_max_> = 3.70 mA cm^−2^) is seen across all materials, suggesting that it does not strictly depend on dimensionality, and should be compared on an individual basis.

Though there is merit in comparing the field emission performance across a single material family as a function of dimensionality, we stress that many materials considered to date do not, as yet, have identified 1D and 2D counterparts. Moreover, the work function of bulk materials can vary dramatically from their nanoscale counterparts. Indeed, the variation in work function between 3D, 2D, and 1D allotropes, as well as within the allotropes themselves, varies dramatically. Nevertheless, the graphitic allotropes offer a prototypical, and importantly, complete dimensional family on which to consider. The graphitic allotropes, including graphite, carbon nanotubes, and graphene, show promising performance. CNTs show a low value of *E*_on_ (1.29 V μm^−1^) compared to the 1D family mean, as well as a high maximum emission current density (6.92 mA cm^−2^ ). Similarly, a mean *E*_on_ of 2.52 V μm^−1^ was recorded for graphene, with a high *J*_max_ of 26.7 mA cm^−2^ compared to the average for this dimensionality. Some materials, such as ZnMgO nanowires (1D) and ZnSe (bulk), show promising performance, with low *E*_on_ of 0.78 V μm^−1^ and 1.40 V μm^−1^, respectively. Nevertheless they exhibit a poor comparison to the average *J*_max_ of their respective dimensionalities (0.35 mA cm^−2^ and 0.63 mA cm^2^, respectively). In contrast to this, Tris(8 hydroxyquinolinato)aluminium (Alq3) nanowires (1D) and WO nanowires (1D) show remarkably high *J*_max_, where Alq3 NWs have a <*J*_max_> of 20.5 mA cm^−2^ and WO NWs show an encouraging value of 13.8 mA cm^−2^ compared to CNTs (6.76 mA cm^−2^). However, Alq3 and WO do not consistently perform similarly well across all metrics, exhibiting higher *E*_on_, with Alq3 NWs showing 9.23 V μm^−1^ and WO NWs with 6.37 V μm^−1^. 2D materials, and in particular the graphenes, show largely similar performance to one another.

The claimed values of *J*_max_ vary significantly within a given material; the data for CVD diamond (polycrystalline)^144^ showed *E*_on_ = 4.42 V μm^−1^, with *J*_max_ = 0.11 mA cm^−2^, while another (which was hydrogen doped)^145^ evidenced *E*_on_ = 58.40 V μm^−1^, and *J*_max_ = 0.01 mA cm^−2^. This results in a standard deviation of 32.4 V μm^−1^ in *E*_on_, which is larger than the mean (21.9 V μm^−1^). There are evidently other, more central factors affecting the field emission capabilities between such cases, than simply the *φ*. Certainly in the present case, the evident doping may affect *φ* marginally, though certainly this would not be to the extent evidenced. With increasing maturity, increased consistency, and increased availability of data, performance metrics collected in this fashion will, in the future, likely reduce this limitation and the values found therein to become more reliable.

In the *φ* ordered materials, no trend is seen on an expected exponential fitting, showing no seeming correlation between *φ* and *E*_on_ or *J*_max_. It is expected, however, that the available data will expand rapidly with the continually emerging and expanding field of nanovacuum electronics, and henceforth a more defined relationship may become apparent. As alluded to earlier, this further supports the notion that there is more to the field emission capabilities of a material than simply *φ* arguments alone, and that other material characteristics have a larger effect on the field emitting capabilities. In combination with other characteristics, such as emitter morphology, and its evident manifestation in *β*, it is plausible that a clearer trend may be noted. Ordering materials in a combined and weighted ranking, such as a combination of *φ* and *β*, in addition to other metrics yet to be identified, may show an improved correlation with the extracted data. In Figure 2, little dependence can also be seen on *J*_max_ with respect to *φ*. This is likely exacerbated by sample‐to‐sample measurement issues, such as minor variations in the measurement systems, as well as the extent to which the voltages of the emitters are driven by different groups. Ongoing systematic studies are underway to investigate the effects of the surface morphology, linked to *β*, on field emission, and the performance metrics, from materials that can be patterned as desired.

There are some factors that are not taken into account that could affect the outcome of the field emission properties of the materials investigated. While, in many reported cases, *φ* is a defined bulk characteristic, surface *φ* of a material can be readily tuned to maximize emission. In practice, surface *φ* is not strictly constant and depends critically on the ambient.^148^, ^149^ The surface *φ* is particularly sensitive to physi‐ and chemisorbed species, with have been implicated in various hysteretic field electron emission studies.^150^ This may well impact on the results from a single material, where otherwise nominally equivalent emitters have been chemically treated differently. In addition, the large data set size will likely induce some statistical scatter, chief amongst which is the length (or height in the case of 2D and bulk materials) of the emitters. This is unlikely, however, to dramatically affect the results, and heights are likely to be within an order of magnitude of one another. Another, potentially more significant factor affecting the emission properties is the cathode fabrication method employed **(**Supporting Information). Such issues may include crystalline damage caused by cleaning processes, such as ultrasonication, different surfactants used in the fabrication process giving variations in dispersion and surface *φ*, as well as experimental conditions such as vacuum, temperature, and pressure levels, driving conditions and applied electrostatic fields, all of which have not been considered herein. The interelectrode spacing may also have an influence on the field emission; however, our data suggest that this is largely negligible (Figure S1, Supporting Information). Just 50% of studies reported the interelectrode distance, *d*. Of those that did, however, *d* had a modal value of 100 μm. 86% of values are within one *σ* of the mean (209 μm), suggesting that the data are largely unaffected by variation in *d*.

Another factor requiring consideration is the fabrication method, which around two thirds of studies stipulate. Due to the extent of the materials used, the number of methods employed reaches 16. There are some materials (such as the CNTs) that can be synthesized using a number of techniques, while other, often newer materials, in general have only a single fabrication method. There is a possibility that this variation, seen amongst those materials that have a number of fabrication methods, results in minor differing field emission behaviour between otherwise equivalent materials though our data suggest this is largely negligible compared to other, more dominant, variations in material parameters. The most common synthesis/fabrication method, however, across all dimensionalities is chemical vapor deposition (CVD), including plasma enhanced and microwave variants. The high numbers of reports using CVD is due largely to the various carbon based materials, which can be grown with desirable features, including alignment and patterning. Similarly, it is possible that in situ or ex situ doping and subsequent variations in the electronic properties of the material occur when CVD and wet chemistry methods are coupled. Even if the fabrication methods are similar, factors such as material composition, lattice configuration, and alignment could all be different and may well affect emission performance dramatically. Nonetheless, the breadth of the study herein was designed to reduce the implications of these varied issues, with the resultant body of evidence indeed supporting our conclusions. Independent studies from different research groups were assessed to form a comparative functional measure across various materials. Though challenging to unify an otherwise disparate field, we have nevertheless, using the present meta‐analysis endeavored to produce the most concise summary to date of all the field emission materials across 1D, 2D, and 3D geometries, consistently evidencing only a very weak dependence on φ in each case. Through the provision of critically compared evidence, the view of the wider field emission community has here been empirically verified in that the surface perturbation and the aspect ratio of the underpinning emitters likely dominate the field emission characteristics over *φ*, with *φ* showing little correlation with enhanced emission. Optimal field emitters will be realized by engineering the interplay between these two critical parameters.

In the present meta‐analysis, we have directly compared the performance of the widest range of field electron emission materials to date. It was found that ordering materials by increasing work function did not result in any clear trend in turn‐on electric field or maximum current density, suggesting other factors must be taken into consideration when discussing the field emitting capabilities of a material. *E*_on_ was found to be twice as large for 3D and bulk materials compared to 1D and 2D materials, suggesting the morphology of the emitter may be significant in regards to determining characteristics effecting field emission. Observations that can be made on a material‐to‐material comparison basis show that few materials seem more promising than the nanocarbons.

## Experimental Section

A digital extraction tool (GetData Graph Digitizer, Vs 2.26.0.20) was used to digitize and gather data from the source metadata using the emission current density, *J*, as a function of the applied electrostatic field, *E*, data sets. In some cases, where current or voltage were given instead of *J* or *E* along an axis, data were converted into the correct form on the condition that the total emitting area or the cathode–anode separation was disclosed. The vast majority of the data (95%), however, was directly extracted from a *J*–*E* curve. All data were replotted and normalized.

The *J*_max_ value represents the maximum current density shown on the graph provided. This may result in variation of definition, whether the tip was run until it failed, array fraction, or maximum current extraction. In most cases, the value is assumed to be represented by the maximum current extracted.

## Supporting information

As a service to our authors and readers, this journal provides supporting information supplied by the authors. Such materials are peer reviewed and may be re‐organized for online delivery, but are not copy‐edited or typeset. Technical support issues arising from supporting information (other than missing files) should be addressed to the authors.

Supplementary
