# Supplementary material for: High Performance Field Emitters
Source: Adv Sci (Weinh). 2016 Feb 18;3(5):1500318. doi: 10.1002/advs.201500318 (PMC5008108; doi:10.1002/advs.201500318)
Supplement: Supplementary file 1 — Supplementary [file ADVS-3-0c-s001.pdf]

Copyright WILEY-VCH Verlag GmbH & Co. KGaA, 69469 Weinheim, Germany, 2015.

ADVANCED SCIENCE
Open Access

Supporting Information

for *Adv. Sci.*, DOI: 10.1002/advs. 201500318

High Performance Field Emitters

*Clare M. Collins, Richard J. Parmee, William I. Milne, and Matthew T. Cole**

High Performance Field Emitters

C. M. Collins,[1] R. J. Parmee,[1] W. I. Milne,[1] & M. T. Cole[1*]

[1] Department of Engineering, Electrical Engineering Division, University of Cambridge, 9 JJ Thomson Avenue, CB3 0FA, Cambridge, UK

*E-mail: mtc35@cam.ac.uk

Supplementary Material

To highlight the inconsistencies in the literature, an exhaustive list of the employed β definitions is given in **Table 1**. Around 70% of the papers quote a β value, regardless of the method they employ to extract β. Table 1 shows the extent of the range used to calculate β. There is a further discontinuity between material types, with 1D and 2D emitters being the most likely to disclose β; over 80% of 1D and 2D papers give β, with a mere 45% stating β in 3D/bulk. 80% of authors stating β use the Fowler-Nordheim slope, whilst 10% used only other methods (shown in Table 1). The remaining number of papers failed to state a method, simply claiming a value of β.

Table 1: Common β definitions

**β – definitions**		Ref.	Geometry
$\beta = (l/r)$	l = emitter length<br>r = emitter radius	[30] [38]	1D/3D
$\beta = (h/r)$	h = emitter height (or length)<br>r = is radius of curvature of tip or radius at apex	[39] [36] [35]	3D
$\beta = \left(\frac{d}{kr_{tip}}\right)$	d = inter-electrode distance<br>k = constant (= 5 for long thin geometry)<br>r = radius of curvature of tip	[143]	1D
$\beta = 100\left(1 + \sqrt{\frac{h}{2r}}\right)$	Smith's model:<br>h = height<br>r = radius of the emitter	[28]	1D
$\beta = \beta_0\beta_s = \beta_0\left[1 - \exp\left(-\frac{cs}{h}\right)\right]$<br>$\beta_0 = 1.2\left(\frac{h}{r} + 2.15\right)^{0.9}$	β_0 = intrinsic field enhancement factor is a ratio of local and macroscopic fields (r is radius).<br>β = overall field enhancement factor,<br>β_s = screening factor<br>s = wire spacing<br>c = constant.	[50]	1D
$\beta = \frac{1}{\left(\frac{h}{d} + \frac{1}{\beta_0}\right)}$	β_0 = enhancement factor (independent of d, h and applied voltage)	[95]	1D
$\beta_0 = \left(\frac{h}{0.95\, r_0}\right)$	β_0 = geometric field enhancement factor<br>h = height<br>r_0 = average radius of tip	[145]	3D
$\beta \approx d^{(1-c)}$	d = inter-electrode distance<br>c = constant (< 1).	[120]	3D

$\beta = -\left(\frac{b\phi^{\frac{3}{2}}}{k}\right)$	b = constant (=6.83 x 10^7 $eV^{3/2}$ V/cm)<br>k = Select gradient of the Fowler-Nordheim plot	All 1D that calculate β	1D, 2D and 3D

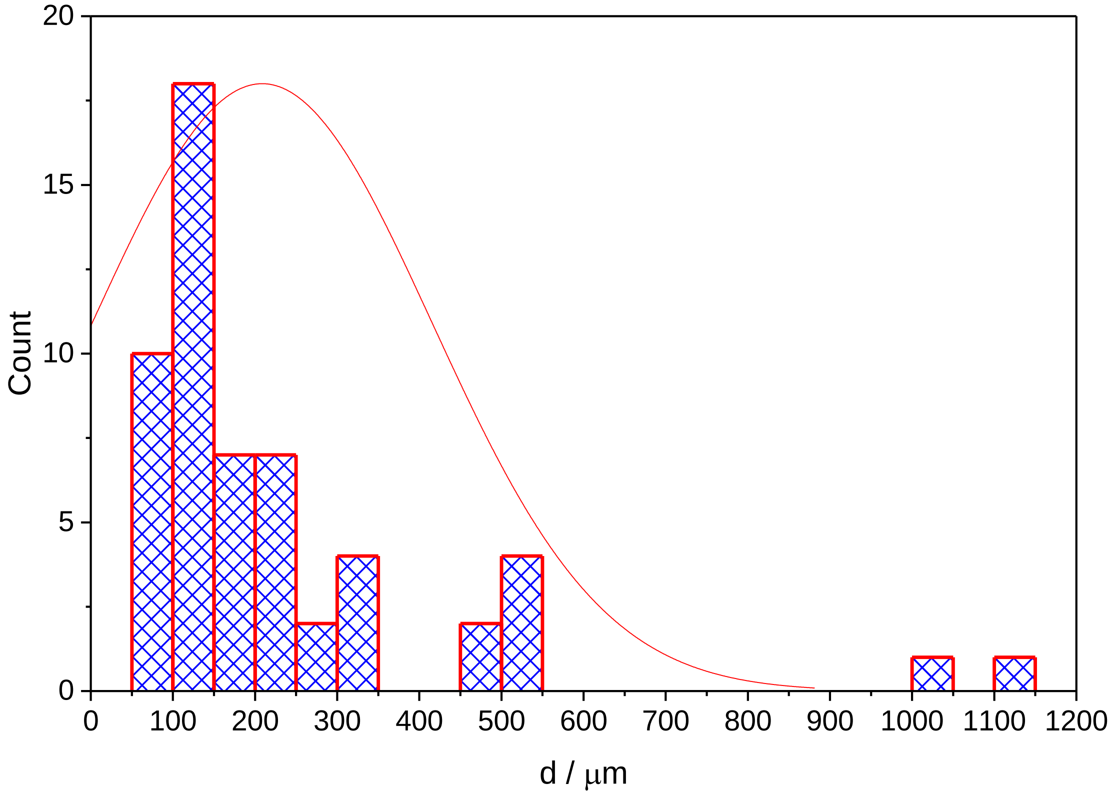

Figure S1. Histogram of the interelectrode spacing (d) of the considered studies.
